# Microhardness evaluation of root dentin after using resin sealer solvents

**DOI:** 10.34172/joddd.2021.042

**Published:** 2021-12-05

**Authors:** Gamze Nalci, Tayfun Alaçam, Bülent Altukaynak

**Affiliations:** ^1^Department of Endodontics, Faculty of Dentistry, Bezmialem Vakıf University, İstanbul, Turkey; ^2^Department of Endodontics, Faculty of Dentistry, Gazi University, Ankara, Turkey; ^3^Department of Statistics, Faculty of Science, Gazi University, Ankara, Turkey

**Keywords:** Chloroform, Ethyl acetate, Methyl ethyl ketone, Solvents

## Abstract

**Background.** This study aimed to assess the effects of methyl ethyl ketone (MEK) and ethyl acetate (EA) on dentin microhardness, used as resin sealer solvents.

**Methods.** Eighty halves of single-rooted teeth were randomly divided into four groups to apply MEK, EA, chloroform, or saline solution. Vickers hardness values were measured for three root levels before and after the direct application of solvents for 5 and 15 minutes or a 1-minute application with ultrasonic agitation. The results were analyzed using repeated-measures ANOVA, and adjustments were made for comparisons with Bonferroni tests.

**Results.** The dentin microhardness decreased in all the solvent groups (*P* < 0.05). The changes in microhardness increased with prolonged exposure times, except for the saline solution group. Chloroform exhibited the most significant decrease in value. Furthermore, ultrasonic agitation elicited a more substantial decline in values.

**Conclusion.** MEK and EA might be preferred over chloroform as a solvent for resin sealers because they offer an attenuated decrease in dentin microhardness and do not have gutta-percha-dissolving properties.

## Introduction

The use of solvents is suggested to simplify the removal of both gutta-percha and sealers. When the endodontic filling is gutta-percha, different solvents can dissolve it in a nonsurgical retreatment procedure efficiently. However, when organic solvents are used, the solvents have more challenges, and resin sealers have an extremely low dissolution capacity to dissolve sealers.^1-3^ Chloroform is the most successful in plasticizing gutta-percha points and sealers and facilitates their removal from the root canal.^4-6^ However, it may only soften the gutta-percha and thus compact the material into the irregularities along the root canal wall and dentinal tubules, after which removal may not be possible.^7^ Ferreira et al^8^ immersed resin material in various solvents and reported that chloroform, ethyl acetate (EA), and methyl ethyl ketone (MEK) exhibited significantly different dissolving profiles for AH Plus sealer compared to xylene, phosphoric acid, and eucalyptol. EA and MEK do not soften gutta-percha; however, they effectively dissolve resin sealer. This property could be the reason for their preferred use in removing resin sealers after gutta-percha has been removed during endodontic retreatment procedures. EA is the ester of ethanol and acetic acid and has many uses, such as artificial fruit essences, aroma enhancers, and artificial flavors for confectionery products, ice creams, and cakes. It is also used as a solvent for many applications, for varnishes and paints, and for the production of printing inks and perfumes.^8,9^ It is classified as a low human health priority and not expected to be potentially poisonous or harmful.^10,11^ MEK is a Food and Drug Administration (FDA)-approved indirect food additive for adhesives and polymers.^12^ In using these solvents, radicular and coronal dentin are exposed to these products, and this contact might cause alterations in dentin microhardness. Thus, it was of interest to investigate to what extent the root canal dentin was affected by using these solvents to dissolve resin sealers.

During smear layer removal, irrigation solutions cause changes in the chemical composition of dentin, which might decrease microhardness and erosion.^13^ Irrigation solutions can affect dentin microhardness and consequently affect the clinical performance of endodontically treated teeth.^14^ Using chelating agents have some advantages, such as cleaning the debris, disinfection, and removal of the smear layer. However, they might cause negative changes in the physical properties of dentin, including altering the microhardness.^15^ A decrease in microhardness can make the instrumentation easier throughout the root canal. However, it might cause a weakened root structure.^16^ Furthermore, a decrease in the microhardness can affect the capability of the sealers to adhere and seal in the root dentin walls.^15^

Besides, ultrasonic tips are efficient for the irrigation of root canals.^17^ Ultrasonic tips rely on the transmission of acoustic energy from an oscillating file or smooth wire to an irrigant in the root canal. The energy is transmitted by ultrasonic waves and can induce acoustic streaming and cavitation of the irrigant.^17,18^

This study aimed to determine dentin microhardness values before and after applying chloroform, EA, MEK, and saline solutions on different levels of root dentin either with a 5- and 15-minute direct contact or application with 1-minute ultrasonic vibration.

## Methods

Forty freshly extracted anterior maxillary teeth with a straight and a single root canal were selected and stored in a 1% thymol solution until used. The teeth were examined under magnification and fiber optic lighting to ensure no cracks or craze lines on the root surface. The teeth were embedded in autopolymerizing acrylic resin using Teflon molds and then sectioned longitudinally in a buccolingual direction into buccal and lingual segments. Sectioning was performed using a low-speed saw (Mecatome, T 2001 A, Pressi, France) under water cooling to produce 80 sections. Pulp tissues were excavated with a dental spoon. For microhardness evaluations, the root canal dentin surface was polished using a circular grinding machine with 400-, 800-, and 1200-grit abrasive papers under running water. Before applying the solutions, Vickers hardness values were measured with a microhardness tester (HMV-700, Shimadzu Corp., Tokyo, Japan) at ×40 magnification with a 200-g load for 15 seconds. Indentations were made with a Vickers diamond indenter at three separate locations for each root canal level (coronal, middle, and apical). The indentations were placed at a distance of 1 mm from the pulp-dentin interface with no overlapping. The microhardness values were recorded as MPa.

**Figure 1:**
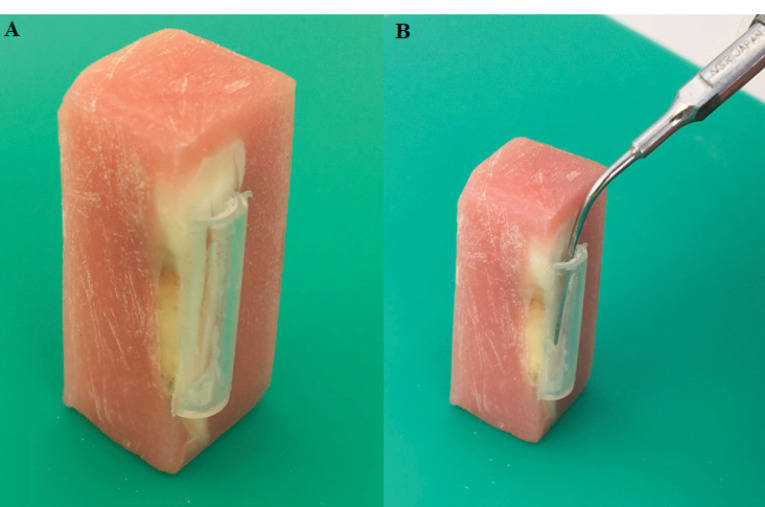
(A) Special designed reservoir; (B) Ultrasonic activation.

Specimens were randomly divided into four groups to apply different solvents, and each group was divided into two subgroups according to the solvent agitation (n = 10 per group). A specially designed reservoir was positioned on the tested root surface and bonded with a light-cured nano-flowable composite resin material (i-Flow N, i-dental, Lithuania; Figure 1A) to apply the solvents. The solvents for this study were: MEK (Kıvanç Kimya, İstanbul, Turkey) in group 1, EA (Kıvanç Kimya İstanbul, Turkey) in group 2, chloroform (BM.2445.1000, Balmumcu Kimya, Ankara, Turkey) in group 3, and sterile saline solution in group 4. The solvents were directly applied to the dentinal surfaces in half of the specimens. The second and third microhardness values were measured after 5 and 15 minutes of successive immersional contact with 2 mL of experimental solution after the subsequent removal and reconstruction of reservoirs. In the other half of each solvent group, ultrasonic agitation was carried out using an ultrasonic device (NSK Various 750 and E8 ultrasonic tip, Nakanishi Inc., Tochigi, Japan) for 60 seconds at 28 kHz (Figure 1B). The ultrasonic tip was placed into the reservoir and agitated using 2 mL of each solution for each specimen. The second microhardness values were then measured. After the treatment, the reservoirs were removed, and all the specimens were rinsed thoroughly with deionized water and dried using absorbent paper.

### Statistical analysis

Continuous normally distributed data were expressed as means and standard deviations (SDs). Changes in pre-test and post-test values were analyzed by paired Student’s *t* tests. The data were analyzed with repeated-measures ANOVA with time and treatment. Post hoc comparisons were performed using Tukey and Bonferroni tests. All the statistics were calculated with SPSS 20.0 (IBM, New York, NY, USA). Differences were considered significant at *P* < 0.05.

## Results

Microhardness values are presented in Tables 1 and 2. Time-related decreases in microhardness were observed in all the solvent groups except saline solution (multiple comparisons: Bonferroni, *P* < 0.05). The strongest effect was observed in the chloroform group, with a time-related decrease in this group (*P* < 0.05). In the MEK group, no differences were found between the initial measurements and the 5-minute application or the 5- and 15-minute applications (*P* > 0.05). However, there was a difference between the initial and 15-minute measurements (*P* < 0.05). In the EA group, there was a difference between the initial and 5- and 15-minute applications (*P* < 0.05); however, the 5- and 15-minute application difference was not significant (*P* > 0.05).

**Table 1:** Time-related microhardness measurements for each solution at each root level for applications with no agitation (n = 30 for each level)

**Solution**	**Time**	**Level**	**Mean** ^¥^	**SD**	**F**	***P*** ** value**
Methyl ethyl ketone	Initial	C	55.06	5.97	1.387	0.255
		M	55.96	5.17		
		A	53.24	7.88		
	5 min	C	57.92^a^	6.50	13.391^**^	0.000
		M	56.25^a^	5.02		
		A	49.18^b^	8.79		
	15 min	C	52.83	3.61	0.205	0.815
		M	52.18	4.39		
		A	52.43	3.93		
Ethyl acetate	Initial	C	60.22^a^	5.95	35.860^**^	0.000
		M	59.20^a^	3.97		
		A	47.36^b^	8.78		
	5 min	C	57.92^a^	4.67	17.484^**^	0.000
		M	50.04^b^	8.51		
		A	44.68^b^	11.58		
	15 min	C	55.66^a^	4.29	15.688^**^	0.000
		M	49.85^b^	5.42		
		A	48.75^b^	5.61		
Chloroform	Initial	C	61.85^a^	11.17	24.465^**^	0.000
		M	65.97^b^	7.32		
		A	50.75^c^	7.05		
	5 min	C	51.67^a^	8.19	11.318^**^	0.000
		M	55.66^b^	4.53		
		A	47.85^c^	5.81		
	15 min	C	51.05^a^	5.25	16.378^**^	0.000
		M	51.87^a^	3.41		
		A	46.08^b^	3.83		
Sterile saline solution	Initial	C	55.06	5.97	1.387	0.255
		M	55.96	5.17		
		A	53.24	7.88		
	5 min	C	56.49	5.34	3.056	0.052
		M	54.76	5.55		
		A	53.05	5.29		
	15 min	C	55.51	6.64	1.030	0.361
		M	54.09	5.85		
		A	53.01	7.71		

C = Coronal, M = Middle, A = Apical; * *P* < 0.05; ** *P* < 0.01; ^¥^Tukey multiple comparisons

**Table 2:** Measurements before and after applying each solution with ultrasonic agitation (n = 30 for each level)

**Solution**	**Ultrasonic**	**Level**	**Mean**	**SD**	**F**	***P*** ** value**
Methyl ethyl ketone	Initial	C	65.05^a^	7.45	29.119^**^	0.000
		M	61.17^a^	7.39		
		A	50.25^b^	8.48		
	1 min	C	47.70	5.89	1.525	0.223
		M	49.43	4.28		
		A	47.06	5.99		
Ethyl acetate	Initial	C	63.32^a^	5.39	42.460^**^	0.000
		M	63.54^a^	5.47		
		A	51.37^b^	6.61		
	1 min	C	48.32	4.59	1.599	0.208
		M	50.17	3.66		
		A	49.15	3.73		
Chloroform	Initial	C	68.26^a^	6.90	35.932^**^	0.000
		M	65.00^a^	7.43		
		A	52.01^b^	9.06		
	1 min	C	47.94^a^	4.11	4.821^*^	0.010
		M	48.90^a^	2.98		
		A	45.84^b^	4.46		
Sterile saline solution	Initial	C	55.06	5.97	1.387	0.255
		M	55.96	5.17		
		A	53.24	7.88		
	1 min	C	57.06^a^	5.04	11.534^**^	0.000
		M	55.30^a^	4.24		
		A	51.54^b^	4.32		

C = Coronal, M = Middle, A = Apical; * *P* < 0.05; ** *P* < 0.01; ^¥^Tukey multiple comparisons.

Comparison of the means showed a difference between chloroform and the MEK and EA groups (*P* < 0.05). There was no difference between MEK and EA groups at 15 minutes (P > 0.05); however, the decrease in microhardness was more significant in the EA group compared to the MEK group at 5 minutes (*P* < 0.05).

There was a difference between the root levels after 5 minutes in the MEK group (*P* < 0.05); however, no difference was found for the 15-minute application (*P* > 0.05). There was a difference between root levels for all time intervals in the EA group (multiple comparisons: Tukey, P < 0.05). There was a difference between the coronal and middle root segments in the MEK and EA groups (multiple comparisons: Bonferroni, *P* < 0.05). The decrease in microhardness in the apical segment was slighter, with no difference between the time intervals for the MEK and EA groups (*P* > 0.05). There was a difference in all the root levels in both time intervals in the chloroform group (*P* < 0.05). The decrease in microhardness in the apical segment was smaller in both time intervals for the EA and chloroform groups (multiple comparisons: Tukey).

The amount of decrease in values was higher when ultrasound was used. There was a difference between the groups (*P* < 0.05), and the difference was more in the MEK group than in the EA group (pairwise comparisons: Bonferroni; *P* < 0.05). However, no effect was registered in the saline solution group (multiple comparisons: Tukey, *P* > 0.05). There was a difference for root segments in the ultrasonically applied MEK and EA groups (*P* < 0.05). For comparisons of changes in root levels with an application of ultrasonic agitation, there was no difference in apical segments in the MEK and EA groups (*P* > 0.05), and there was a time-dependent difference in the coronal and middle segments in the MEK and EA groups (*P* < 0.05). There was a difference in all the root levels after the application of ultrasound in the chloroform group (*P* < 0.05); however, the difference in the apical segment was less than that in the coronal and middle root segments.

## Discussion

In this study, surface changes in dentin, after the use of MEK and EA, were demonstrated, and the values were compared with chloroform using microhardness testing. Previous studies have shown the convenience and practicality of using Vickers microhardness tests for evaluating surface changes in dental hard tissues treated with various chemical solutions.^10^ Microhardness value can be an indirect indicator of mineral loss or deposition in dental hard tissues.^19^ Moreover, a positive correlation exists between the microhardness values and the mineral content of teeth.^20^

Different solvents have various effects on different sealers, and resin sealers are nearly insoluble by many solvents.^2,10,21-23^ Chloroform is a very effective chemical in dissolving gutta-percha and sealers. Whitworth and Boursin^6^ found that a 10-minute application of chloroform dissolved 96% of an AH Plus (Dentsply De Trey GmbH, Germany) epoxy resin sealer. Similar results were reported by Martos et al.^9^ However, Erdemir et al^10^ tested the solvent action of chloroform on completely set AH 26 and AH Plus materials in capillary glass tubes and observed that solvents were not effective in removing sealers from the tubes within 30 minutes. Combined with its toxicity risk, the possible obliteration of tubule openings by the precipitation of gutta-percha might be another problem in subsequent procedures by the clinical use of chloroform.

The use of solvents, other than chloroform, that cannot dissolve gutta-percha, like EA and MEK, might be reasonable for removing resin sealers in clinical settings. Ferreira et al^8^ reported EA was comparable to chloroform for removing an epoxy resin sealer without any potential hazards. Besides, MEK showed successful activity in their study at 5 minutes. However, solvents used for dissolving resin sealers might change the physical and chemical properties of dentin, and this issue might be clinically relevant because alterations in the dentin surface might affect dentin’s interaction with materials used for obturation.

This study aimed to evaluate the effect of these chemicals on the microhardness of dentin. There is a wide variation of microhardness values after applying endodontic solvents in the literature. Erdemir et al^10^ found that chloroform and halothane did not affect the microhardness or roughness of root dentin (*P* > 0.05). On the other hand, another study concluded that using chloroform, xylene, and halothane as chemical solvents for more than 5 minutes during the treatment significantly decreased the microhardness of enamel and dentin.^24^ It was presumed that the gutta-percha solvents might cause alterations of the organic components of enamel and dentin by enlarging the intercrystalline spaces and increasing the porosity and permeability of enamel and dentin tissues. Erdemir et al^10^ exposed dentin to 20 mL of solvents for 15 minutes. In the present study, in the non-agitated application group, dentin was exposed to 20 mL of solvent for up to 15 minutes. Since there is no standard model for performing tests, variations might occur in studies. Therefore, the researchers established the materials, the time interval, temperature, contact surface on which the solvent would act, and the device used to measure the results. This study indicated that all the chemical agents, except saline solution, significantly decreased the microhardness of root dentin (*P* < 0.05). Among the test groups, chloroform caused the maximum reduction in microhardness of dentin. Thus, MEK and EA might be preferred over chloroform for retreatment cases when resin-based sealers are used since the decreases in dentin microhardness are attenuated with MEK and EA.

In a study by Ballal et al,^25^ indentations were made 0.5 mm from the canal wall. However, in Akçay et al’s^26^ study with Vickers test for microhardness measurements, indentations were made in the middle third of the root canal, 100 μm from the pulp-dentin interface. In a study by Ballal et al,^25^ a 200-g load with 20 seconds of dwell time was used. However, in another group, a 50-g load was applied for 15 seconds to each specimen.^27^ Thus, in the current study, we preferred using a milder load with a shorter duration; therefore, our indentations were made at 100 μm from the root canal surface with a 50-g load applied for 15 seconds.

Passive ultrasonic activation was applied with different substances as an auxiliary method to improve gutta-percha and sealer removal. During the retreatment, ultrasonic agitation of organic solvents can be beneficial for enhancing the chemical activities of these substances, and hence, they increase their dissolving capacities for root canal sealers. In another study, hand instrumentation/chloroform and ultrasonic/hand instrumentation/chloroform were compared to remove gutta-percha associated with AH 26, Roth’s 801, and Ketac Endo.^28^ As a result, it was found that the ultrasonic method is faster than hand instrumentation. Ferreira et al^8^ also found that ultrasonic activation of solvents gives rise to a significant increase in the mean dissolution of the AH 26. However, Trevisan et al^29^ found that the solvent and different times did not affect the weight loss of the resin sealer. The ultrasonic technique with chloroform, EA, and MEK had more profound effects than the same solvents applied with no agitation in the current study, as expected.

Moreover, these results could be achieved in a shorter time with ultrasonic instrumentation. Agitation of the solution and frictional heat generated by the ultrasonic tips might increase the solvent action, reducing the time required for sealant removal. Ultrasound energy was applied in the current study at the maximum intensity for only 60 seconds as specified by Sabins et al.^30^

In the present study, chloroform induced the most significant reduction in root dentin microhardness at all three root levels, possibly because of its better demineralizing effect owing to its high acidity. There was no difference between MEK and EA, especially at the apical level, and the difference in chloroform can be explained by the lower pH compared to other solutions. Only one study compared the effects of MEK, EA, and chloroform on root dentin microhardness. However, in this study, the differences between the root levels were not specified. Our study’s finding is consistent with that of Ferreira et al,^8^ who reported that dentin microhardness was significantly reduced by chloroform, EA, and MEK, respectively.

All the solvents and application methods reduced hardness at all three levels. However, the least amount of reduction was at the apical level, which might be attributed to the histological structure of the apical region. The ratio of the organic matrix decreases in the apical part of the root dentin, which might explain the lower degree of decalcification in this part of the root.^31^ Additionally, the apical part of the root canal might not receive proper irrigation as it is anatomically narrow.

Improvements in materials and methods for dissolving gutta-percha and sealers without harmful effects are the desired outcome in endodontic retreatments. Highly reactive solvents and agitated auxiliary methods could be more hazardous for dental hard tissues and periradicular tissues if handled incorrectly. In considering the removal of resin cements, the choice of EA or MEK would be preferable over chloroform since they have a milder harmful potential on the dentin.

## Authors’ Contributions

All authors have made substantive contributions to planning and executing this study, and all have reviewed the final paper before its submission and publication.

## Acknowledgments

None.

## Funding

None.

## Competing Interests

The authors deny any conflicts of interest related to this study.

## Ethical Approval

This study was approved by the Ethics Committee of Ankara University Faculty of Dentistry, Turkey (Reference number: 36290600/79).
